# The Association Between Herpes Zoster, Antiherpetic Therapies, and Alzheimer’s Disease: A Comprehensive Systematic Review

**DOI:** 10.3390/ph18050722

**Published:** 2025-05-14

**Authors:** Ali Alghamdi, Emma Koen, Manon Helmantel, Karim Rafie, Amalia M. Dolga, Barbara C. van Munster, Eelko Hak

**Affiliations:** 1 Groningen Research Institute of Pharmacy, PharmacoTherapy, -Epidemiology & -Economics, University of Groningen, 9713 AV Groningen, The Netherlands; 2 Department of Pharmacology and Toxicology, Faculty of Pharmacy, King Abdulaziz University, Jeddah 21589, Saudi Arabia; 3 Department of Molecular Pharmacology, Groningen Research Institute of Pharmacy, University of Groningen, 9712 CP Groningen, The Netherlands; 4 Department of Geriatric Medicine, University Medical Center Groningen, University of Groningen, 9700 AB Groningen, The Netherlands; 5 Alzheimer Center Groningen, University Medical Center Groningen, 9713 GZ Groningen, The Netherlands; 6 Department of Epidemiology, University Medical Center Groningen, University of Groningen, 9713 GZ Groningen, The Netherlands

**Keywords:** Alzheimer’s disease, herpes zoster, varicella zoster virus, antiherpetic drugs, vaccination

## Abstract

**Background:** Alzheimer’s Disease (AD) represents a significant public health challenge with a rising global burden. Emerging evidence suggests a potential link between herpes zoster (HZ) and an increased risk of AD. These findings indicate that HZ may contribute to neuroinflammation and other pathophysiological processes associated with AD, pointing out the potential role of the virus infection in the initiation and/or progression of AD pathology. **Objective:** This systematic review evaluates the relationships between HZ and the risk of AD, highlighting the potential neuroprotective role of HZ vaccination and antiherpetic drug (AHD) use. **Methods:** Adhering to the (Preferred Reporting Items for Systematic Reviews and Meta-Analyses) PRISMA guidelines, a comprehensive search of databases (e.g., PubMed) until October 2024 was conducted. Observational studies reporting risk estimates were included. Extracted data were synthesized and analyzed narratively due to study heterogeneity. **Results:** Eleven studies (out of two hundred) met the inclusion criteria, offering preliminary insights that could form a foundation for further investigation. Reports on HZ showed mixed outcomes, with some studies suggesting a potential increased risk of AD, while others showed no clear correlation. Interestingly, HZ vaccination led to a potential preventive role in reducing the risk of AD, with risk estimates ranging from 0.57 to 0.99. Additionally, the use of AHDs was linked to a reduced risk of AD, with risk estimates ranging from 0.65 to 0.96. **Conclusions:** Published findings suggest that HZ vaccination and AHD use could represent potential interventions to reduce the risk of AD; however, further research is necessary to validate these findings and better understand the underlying protective mechanisms.

## 1. Introduction

Alzheimer’s Disease (AD) is considered to contribute the most to the incidence of dementia, with a contribution of 60 to 80 percent of dementia cases. AD is a neurodegenerative disorder that progresses slowly. This damage often starts in the entorhinal cortex, a part of the medial temporal lobe, and frequently spreads to the hippocampus, a critical brain region for memory and behavior. Both areas are heavily affected, leading to cognitive decline and behavioral changes [1]. AD represents a global public health problem, affecting over approximately 55 million people worldwide, with the number being expected to increase to 139 million by 2050 due to increasing population growth and aging [2] (accessed on 3 January 2025). This highlights the urgent need for preventive and therapeutic interventions to reduce the risk of AD.

The etiology of AD is multifactorial, involving genetic predisposition, environmental exposures, and lifestyle factors [3]. Viral infections, particularly those caused by neurotropic herpesviruses, such as varicella-zoster-virus (VZV), have recently been implicated in AD pathogenesis [4,5,6]. The reactivation of VZV can induce systemic and neuroinflammatory responses, exacerbating neurodegenerative processes, including amyloid beta (Aβ) aggregation and tau protein hyperphosphorylation, which are all hallmark features of AD pathology [7,8]. Recent evidence suggests that the reactivation of VZV may contribute to the pathogenesis of AD through multiple interconnected pathways involving neuroinflammation and neuronal dysfunction. VZV-induced inflammation can activate microglia and lead to the release of proinflammatory cytokines, promoting chronic neuroinflammatory states associated with synaptic loss and neurodegeneration [9,10]. Experimental and early in vivo studies suggest that VZV may contribute to AD pathology by promoting Aβ accumulation and tau phosphorylation. In vitro, VZV infection of human astrocytes and brain vascular cells induced Aβ and amylin buildup and activated neurodegenerative gene pathways [11]. A postmortem analysis of a VZV encephalitis case revealed intracellular Aβ and tau tangles, though they were not consistently co-localized with viral presence, suggesting indirect effects [12]. Additionally, VZV has been shown to reactivate latent Herpes simplex virus type 1 (HSV-1) which then drives Aβ and p-tau pathology, pointing to a possible synergistic viral mechanism in AD development [13]. In parallel, the accumulation of Aβ, a key hallmark of AD, may be triggered as part of the brain’s innate immune response to viral pathogens—a mechanism described by the antimicrobial protection hypothesis [9]. Moreover, VZV infection has been shown to induce tau hyperphosphorylation and disrupt amyloid precursor protein processing, further contributing to AD-like neuropathology [10]. These effects are exacerbated by age-related immune decline, chronic antigenic stimulation, and compromised blood–brain barrier integrity, which together facilitate the entry of inflammatory mediators and viral components into the central nervous system [9].

Preventive strategies targeting VZV infections, including vaccination and antiherpetic medications, have gained attention for their potential neuroprotective effects. The herpes zoster vaccine may reduce the risk of AD by boosting VZV-specific T-cell responses, maintaining viral latency, and limiting inflammatory reactivations that promote amyloid and tau pathologies. Additionally, live-attenuated vaccines like the zoster vaccine may exert off-target immune effects, enhancing long-term immune regulation and reducing systemic inflammation in aging [14,15]. Recent quasi-experimental studies from Australia and Wales showed that herpes zoster vaccination significantly reduced the dementia risk, suggesting that both antiviral and broader immunomodulatory mechanisms contribute to its protective effect [14,15]. Vaccination against VZV, particularly with recombinant herpes zoster vaccine (RZV), has been shown to significantly reduce the incidence of shingles and its complications [16,17]. Antiherpetic drugs, such as acyclovir, valacyclovir, and famciclovir, are widely used to manage VZV infections and suppress viral replication, potentially mitigating the neuroinflammatory effects of herpesvirus reactivation and reducing the risk of AD [18,19]. AHD may offer neuroprotection by suppressing herpesvirus reactivation in the brain, thereby reducing virus-induced amyloid-beta accumulation and tau phosphorylation [13]. These medications also limit chronic neuroinflammation by preventing viral replication, which helps mitigate microglial activation and neuronal damage [18].

The limited effectiveness of current therapeutic options for AD, along with the disappointing results from traditional drug discovery projects using experimental animal models, underscores the urgent need for a different approach [20]. This has led to the initiation of the Drug Repurposing for Effective Alzheimer’s Medicines (DREAM) study, introducing an alternative multidisciplinary approach using an omics-based methodology [21]. This innovative study aims to retrieve drug repurposing candidates for AD and related dementias (ADRD), based on the hypothesis that AD is a multifactorial brain disorder. Studies suggest that elevated tumor necrosis factor (TNF) levels, triggered by a VZV infection, can act as a trigger for heightened neuroinflammation, linking them to AD pathogenesis [21]. Additionally, infection with VZV is common in adults over the age of 50, and preventive strategies like vaccination and antiviral therapies are widely available.

This review addresses a key research gap by systematically evaluating the associations between VZV infection, VZV vaccination, and AHD use with the risk of AD. Unlike prior frameworks such as the DREAM study, which broadly link infections to neurodegeneration, this review uniquely integrates evidence on infection, prevention, and treatment that is specific to HZ. By combining these exposures, it offers a new perspective on viral pathways in AD pathogenesis and highlights opportunities for preventive interventions.

In this review, HZ specifically refers to the reactivation of VZV in the brain or central nervous system (CNS), which may contribute to neuroinflammatory processes that are implicated in AD pathogenesis.

## 2. Methods

This review adhered to the PRISMA (Preferred Reporting Items for Systematic Reviews and Meta-Analyses) guidelines [22]. The PubMed^®^ database was searched from the start of the database until 27 February 2024. The used search strategy was as follows: (“Alzheimer Disease” [Mesh] OR “Dementia” [Mesh]) AND (“Herpes Zoster” [Mesh] OR “Herpesvirus 3, Human” [Mesh]). Also, the references of the included studies were studied to find relevant articles for the study. Human epidemiological studies published until October 2024 were included.

## 3. Eligibility Criteria

Analytical observational studies, including cohort, case–control, and cross-sectional studies, examining the associations between VZV infection, vaccination, or AHD use and AD outcome (with a clear distinction that the outcome is AD and not general dementia) were included. Additionally, randomized controlled trials (RCTs) investigating these associations were also sought, although none met the inclusion criteria. Eligible studies reported risk estimates, such as odds ratios (ORs), relative risks (RRs) or hazard ratios (HRs) with their corresponding 95% confidence intervals (95% CIs). It is important to note that ORs and HRs derived from different study designs are not directly comparable due to differences in underlying assumptions, time frames, and analytical methods. The search was limited to human epidemiological studies published in English up to October 2024, with no restrictions on the publication date for the initial search. Exclusion criteria included studies without risk estimates, preclinical research, narrative reviews, meta-analyses, case reports, and studies lacking full-text availability.

## 4. Data Extraction

Key data, including the study design, geographic region, population characteristics, sample size, exposures, outcomes, and risk estimates, were extracted independently by three reviewers (A.A., M.H., and E.K.) to ensure the accuracy of data extraction and registration. If there was any disagreement, it was resolved through consultation with E. Hak. Due to heterogeneity in study designs and methodologies, a narrative synthesis was conducted. The results were stratified into three categories: (1) VZV infection and AD risk, (2) VZV vaccination and AD risk, and (3) AHD use and AD risk.

## 5. Results

**Figure 1:**
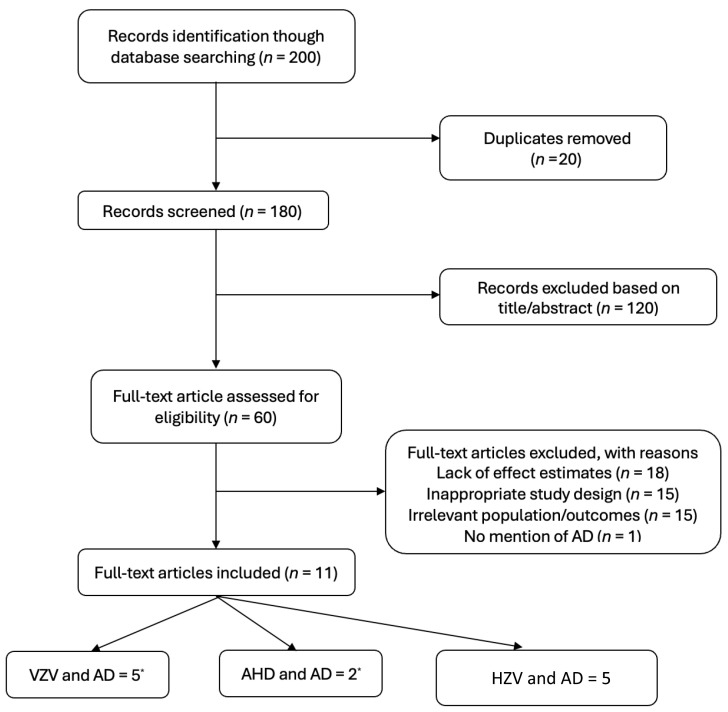
Flow chart of the inclusion and exclusion of papers. (*) some of these studies have two exposures. VZV = varicella zoster virus; AHD = antiherpetic medication; HZV = herpes zoster vaccination; AD = Alzheimer’s Disease.

A total of 200 records were identified from database searches. After removing 20 duplicate records, 180 unique articles remained (Figure 1). The title and abstract screening excluded 120 studies for failing to meet the inclusion criteria. The full-text screening of 60 articles led to the exclusion of 49 studies for the following reasons: (1) a lack of effect estimates (*n* = 18); (2) inappropriate study designs, including narrative reviews and case reports (*n* = 15); (3) irrelevant populations or outcomes (*n* = 15). The outcome was only dementia without AD (*n* = 1). Eleven studies ultimately met the inclusion criteria and were synthesized for this review. These studies explored the relationships between VZV infection [10,18,23,24,25,26], HZ vaccination [27,28,29,30,31,32], and AHD use [19,24] with the risk of AD (Table 1).

### 5.1. Varicella Zoster Virus Infection and Alzheimer’s Disease

Several studies have explored the potential link between VZV infection and an increased risk of AD, but their findings are inconsistent, making the overall evidence inconclusive. In a large population-based cohort study in South Korea, Bae et al. investigated the relationship between VZV infection and AD, as well as the effect of antiviral therapy on the risk of AD. Among 229,594 individuals aged ≥50 years, VZV infection was associated with an increased risk of AD [adjusted HR (aHR): 1.12; 95% CI: 1.04–1.19]. Notably, patients who received antiviral treatment had a lower risk of AD compared to untreated patients (aHR: 0.79; 95% CI: 0.69–0.90) [18]. In a nationwide cohort study involving 371,197 individuals aged ≥50 years in South Korea, Shim et al. reported a significant association between HSV and VZV infections and AD. HSV infections were linked to an 18% increased risk of AD (aHR: 1.18; 95% CI: 1.16–1.20; *p* < 0.001), while VZV infections increased the risk by 9% (aHR: 1.09; 95% CI: 1.07–1.11; *p* < 0.001) [26]. In contrast, Choi et al., after analyzing over 11,000 AD cases and 45,000 controls, found no association between VZV infections and AD (adjusted OR: 0.90; 95% CI: 0.84–0.97) [23]. These findings remained consistent across different age groups and sexes, indicating no increased risk of AD following a VZV infection.

Consistent with these findings, Schmidt et al. observed that over a median follow-up of six years, the adjusted hazard ratio (aHR) for AD in HZ patients was 0.98 (95% CI: 0.92–1.04) during the first year and 0.93 (95% CI: 0.90–0.95) thereafter. AD was diagnosed in 9.7% of HZ patients compared to 10.3% of controls, suggesting that VZV vaccination is unlikely to significantly reduce the AD incidence [25].

In a nationwide matched cohort study in Sweden, Lindman et al. examined the relationship between herpesvirus infections (HSV-1 and VZV), antiviral treatment, and dementia, including AD, by observing 265,172 individuals aged ≥50 years. The study found that untreated herpes infections were associated with a 50% increased risk of AD (aHR: 1.50; 95% CI: 1.29–1.74), while antiviral treatment was linked to an 11% reduction in the risk of AD (aHR: 0.89; 95% CI: 0.86–0.92) [24] (Table 2).

### 5.2. Varicella Zoster Virus Vaccination and Risk of Alzheimer’s Disease

All published studies evaluating the association of VZV vaccination with dementia and AD consistently showed reduced risks of AD in vaccinees versus the reference groups. Harris et al. analyzed data from adults aged ≥65 years over an 8-year follow-up and found VZV vaccination significantly reduced the risk of AD. Vaccinated individuals had a lower AD incidence (8.1%) compared to unvaccinated individuals (10.7%), with a relative risk (RR) of 0.75 (95% CI: 0.73–0.76) and a stronger protective effect for the recombinant VZV vaccine (Shingrix^®^; RR = 0.27) than the live-attenuated vaccine (Zostavax^®^; RR = 0.92) [31]. Scherrer et al. conducted a retrospective cohort study using data from the Veterans Health Administration (VHA) and MarketScan databases, involving individuals aged 65 and older without prior dementia. VZV vaccination, primarily involving a single dose of Zostavax, was significantly associated with a reduced risk of AD, with an aHR of 0.75 (95% CI: 0.71–0.80) in the VHA cohort and 0.70 (95% CI: 0.55–0.88) in the MarketScan cohort [29]. Similarly, Lophatananon et al. (2023) [28] analyzed data from 854,745 vaccinated and 8.8 million unvaccinated individuals aged >70 in England. The vaccination involved a single dose of **Zostavax**, as Shingrix had not yet been introduced into routine practice during the study period. Their results showed a significant reduction in the risk of AD (HR: 0.91; 95% CI: 0.89–0.92), supporting the potential protective role of the vaccine [28]. Schnier et al. examined 336,341 individuals in Wales using Secure Anonymised Information Linkage (SAIL) databank records. Most participants received a single dose of **Zostavax**, and the results indicated a reduced risk of AD following vaccination (aHR: 0.81; 95% CI: 0.77–0.86) [30]. Lehrer and Rheinstein analyzed data from the Behavioral Risk Factor Surveillance System (BRFSS), which included adults aged 60 and older in the United States. The study evaluated the association between Zostavax and AD, focusing on cognitive outcomes. The results showed a 15% reduction in dementia risk among vaccinated individuals and a statistically significant lower likelihood of cognitive decline interfering with social activities in vaccinated compared to unvaccinated participants [27] (Table 3).

### 5.3. Antiherpetic Drug Use and Alzheimer’s Disease Risk

Several studies showed that the use of AHDs was associated with a reduced risk of AD [19,24]. Lindman et al. conducted a registry-based cohort study in Sweden involving 265,172 individuals aged ≥50 years who were diagnosed with HSV or VZV infections or prescribed antiviral drugs, along with matched controls. Over a follow-up period of up to 12 years, untreated herpes infections were associated with an increased risk of AD (aHR = 1.50; 95% CI: 1.29–1.74), while antiviral treatment reduced this risk (aHR = 0.89; 95% CI: 0.86–0.92). The AD incidence rate was 12.9 per 1000 person-years for untreated herpes cases versus 8.5 for treated cases. The most commonly prescribed AHD medications were valacyclovir and acyclovir, which are typically administered at doses of 1000 mg three times daily for 7 days and 800 mg five times daily for 7 days, respectively, according to Swedish clinical guidelines. Other antiviral agents used included ganciclovir, famciclovir, cidofovir, and valganciclovir. A longer duration of antiviral treatment (>30 days) showed a stronger protective effect against AD [24].

Similarly, Linard et al. found a reduced risk of AD among individuals using antiherpetic drugs (AHDs) (HR = 0.85; 95% CI: 0.75–0.96). The most frequently prescribed AHDs were valacyclovir and acyclovir, administered at doses of 1000 mg three times daily for seven days and 800 mg five times daily for seven days, respectively, as per French treatment guidelines [19] (Table 4).

## 6. Discussion

This systematic review highlights the complex relationship between varicella zoster virus infections and Alzheimer’s Disease (AD). While the evidence regarding the direct association between VZV infection and AD remains inconclusive due to study heterogeneity and methodological variations, it underscores the need for further high-quality research in this area. On the other hand, VZV vaccination consistently demonstrated a protective effect against the risk of dementia, including AD, suggesting that vaccination may play a significant role in reducing neurodegenerative risk by preventing viral reactivation. Additionally, the articles discussing the use of antiherpetic medications in relation to AD consistently reported a reduced risk among individuals who were treated with systemic antiherpetic drugs. Both studies indicated that suppressing herpesvirus reactivation through antiviral therapy, primarily with valacyclovir and acyclovir administered in short courses according to clinical guidelines, may offer neuroprotective benefits. Despite these promising findings and as per our inclusion criteria, only two studies were identified, highlighting the need for more extensive research to confirm these observations, assess the long-term effects, and explore the underlying mechanisms of antiherpetic medications in AD prevention.

### 6.1. Varicella Zoster Virus Infection and AD Risk

The association between VZV infection and the risk of AD varied across studies. Bae et al. found an HR of 1.12, indicating a 12% increased risk of AD among those with VZV, which was attributed to neuroinflammation and systemic immune activation induced by viral reactivation [18]. In contrast, Schmidt et al. reported an HR of 0.93, suggesting a 7% decreased risk, which was potentially confounded by antiviral therapy in their study population [25]. These protective associations may reflect differences in antiviral drug exposure, possibly by preventing viral reactivation and subsequent neurodegeneration. Demographic data like age at infection and genetic differences may influence whether VZV is a negative factor in cognitive decline. These conflicting findings highlight the need for future studies to account for treatment effects and population variability and provide consistent definitions of VZV exposure and AD outcomes.

### 6.2. Varicella Zoster Virus Vaccination and AD Risk

The protective effects of VZV vaccination were consistently observed across multiple studies. Both Zostavax and RZV demonstrated significant reductions in the risk of AD, with the latter showing greater efficacy and longer duration of protection. Schmidt et al. reported that RZV is administered in a two-dose schedule, with doses given 2 to 6 months apart. Each dose contains 50 µg of varicella-zoster virus glycoprotein E antigen, combined with an adjuvant to boost the immune response, providing long-lasting protection against herpes zoster and its complications [25]. Harris et al. reported an RR of 0.27 for Shingrix, indicating a 73% reduction in the risk of AD, and 0.92 for Zostavax, reflecting an 8% reduction [31]. Scherrer et al. found HRs ranging from 0.70 to 0.75, pointing to a 25–30% risk reduction [29]. The hypothesized mechanism involves the prevention of viral reactivation and the subsequent reduction in neuroinflammatory responses [13,33].

### 6.3. Antiherpetic Drug Use and AD Risk

AHD use emerged as a promising intervention for reducing the risk of AD. Lindman et al. reported an HR of 0.89 (11% risk reduction), while Linard et al. found an HR of 0.85 (15% risk reduction), suggesting that suppressing herpesvirus reactivation may mitigate neuroinflammation and subsequent neurodegenerative processes [19,24]. Molecular studies have corroborated these findings, indicating that antiviral therapies may directly counteract neuropathological effects associated with herpesvirus infections [33]. However, the observational nature of these studies limits the ability to draw definitive causal inferences.

The findings of this review underscore the need for robust longitudinal studies and RCTs to address the methodological limitations observed in the existing research. Future studies should focus on standardizing the definitions of VZV exposure, vaccination, and AD outcomes while adjusting for confounding variables such as genetic predispositions, socioeconomic factors, and comorbidities. Investigating optimal vaccination schedules and antiviral treatment protocols could provide actionable insights into AD prevention strategies. Expanding the evidence base through high-quality studies will enable stronger causal inferences and enhance the applicability of these interventions in public health contexts.

## 7. Policy Implications

Although VZV vaccination and AHD use appear promising for Alzheimer’s Disease prevention, practical barriers remain. Vaccine uptake among older adults is often limited by hesitancy, accessibility issues, and safety concerns. Public health strategies should prioritize increasing awareness about the cognitive benefits of VZV vaccination and integrating recombinant zoster vaccine (RZV) into routine immunization programs for older adults. Also, facilitating access to antiviral therapies for individuals at risk could represent another preventive opportunity. Cost-effectiveness analyses have shown that preventive strategies addressing AD risk factors, such as VZV vaccination, are economically beneficial [34]. Additionally, the integration of AHDs into AD prevention protocols warrants further investigation through well-designed RCTs to optimize their therapeutic potential.

## 8. Limitation

The findings of this review should be interpreted with caution due to several inherent limitations. Most included studies were observational in nature and thus susceptible to confounding, surveillance bias, and residual confounding. Although many studies adjusted for known confounders, unmeasured variables such as socioeconomic status, baseline cognitive function, and healthcare-seeking behavior may have influenced the observed associations. Surveillance bias is also a concern, as individuals who were vaccinated or treated may have had more frequent healthcare interactions, potentially leading to earlier dementia diagnoses.

Moreover, the heterogeneity in study methodologies and populations further complicates interpretation. While the identified associations are promising, they cannot be considered causal without confirmation from RCTs or other rigorous study designs. Longitudinal studies and well-designed RCTs are essential to establish causality and guide evidence-based preventive strategies.

Another limitation relates to missing data. Although most studies [18,25,26] reported low levels of missing outcome data—likely due to the use of administrative or health insurance databases—few specified the extent of missing covariate data or whether imputation techniques were applied. Importantly, none of the studies conducted sensitivity analyses to evaluate the robustness of their findings under different assumptions or data definitions.

Finally, our review was limited by the lack of mechanistic data. Information on biomarkers, inflammatory markers, neuroimaging findings, or detailed CNS involvement was not available in the included studies. Such data are crucial to improving the biological plausibility of the observed associations. Future research should aim to incorporate these mechanistic factors to support causal inference and elucidate underlying pathways.

## 9. Critical Appraisal of Reviewed Studies According to the DREAM Study

This systematic review aimed to clarify the association between VZV infection and the risk of AD development by the assessment and inclusion of relevant articles. The study findings propose that (1) inconsistency exists concerning the association between VZV infections and AD; (2) VZV vaccinations might reduce the risk of AD; and (3) AHD intake might exhibit a protective role in mitigating the risk of AD. However, a critical appraisal of studies is required to minimize bias and provide the best available scientific evidence. The DREAM study already introduced this topic, describing key threats to validity that are inherent to pharmacoepidemiologic studies and presenting solutions to overcome them. Most of the limitations they outlined also emerged in several studies incorporated in this systematic review, affecting the reliability of the studies [21]. The main possible flaws in design or analysis are given below, provided with some examples.

### 9.1. Misclassification Bias

Misclassification bias refers to the incorrect categorization of study participants into a particular category, resulting in inaccuracies during the investigation of a specific association. Two main forms of misclassification highlighted in the DREAM study are exposure and outcome misclassification. Both misclassification errors came across in a variety of the reviewed studies. For example, it appears to be a challenge to distinguish the type of herpesvirus when identifying VZV-exposed patients based on prescriptions due to similar treatment regimens among different herpesviruses, potentially introducing exposure misclassification. Similarly, diagnoses of ADRDs are prone to outcome misclassification, as these disorders manifest slowly and patients may not be diagnosed during follow-up. In addition, outcome misclassification among the different types of ADRD might occur. Therefore, the DREAM study emphasized that all pharmacoepidemiologic studies investigating the association between a specific treatment and ADRD risk are subject to outcome misclassification bias, albeit to varying degrees. If such misclassification occurs randomly, it may lead to a null effect or underestimation of the risk estimate.

### 9.2. Selection Bias

Another validity error that may lead to invalid results from epidemiological studies is selection bias. This occurs if there is a mistake during the selection of study participants that prevents the study sample from being representative of the source population regarding exposure or outcome. Lindman et al., amongst others, mentioned that their herpes diagnoses came exclusively from special clinics, which most likely resulted in a study sample comprising more fragile individuals compared to the average herpes-infected patient [24]. This may lead to overestimations of the risk of AD in this group as opposed to the unselected reference group. In addition, all studies evaluating the association between VZV vaccination and the risk of AD development reported selection bias as a potential limitation [27,28,29,30,31,32]. A specific type of selection bias was highlighted: the “healthy vaccinee” bias. This specifically refers to a situation where the study sample of vaccinated persons consists primarily of individuals who are generally healthier than the overall population. Correspondingly, Schnier et al. indicated that their findings might be distorted, since individuals being vaccinated generally have a higher and healthier life expectancy and thus might have a reduced risk of developing AD [30].

### 9.3. Confounding Bias

A third systematic error is the potential presence of confounding variables that might affect the association between determinant and outcome variables. Depending on the specific study of concern and its subject matter, a wide variety of different confounders might be present. To prevent a distorted association, it is essential to adjust for these variables, if measured. Although the studies in this systematic review attempted to control for the majority of potential confounders, they mentioned the likely persistence of unmeasured factors, often referred to as residual confounding. For example, the articles from Choi et al. and Shim et al. highlighted that no data were provided on possible confounders, including alcohol consumption, smoking history, body mass index, and education [23,26]. As a result, this inability to adequately account for these potential confounders might have introduced bias into their analysis [23,26].

Additionally, another confounder was identified in studies assessing the association between VZV infection and the risk of AD [23,25]. In this case, diagnosis based on antiviral treatment was identified as a potential confounding factor, given its suggested potential to affect both VZV infection and AD onset. Strikingly, two out of three studies that did not find VZV infection to be associated with an increased risk of AD selected their VZV-exposed participants primarily based on prescriptions with VZV-specific antiviral medications. As a consequence, their unexpected outcome where VZV infection seemed to result in a decreased risk of AD might be distorted by a possible protective role of these therapeutics, as previous research suggested [23,25].

### 9.4. Causality Bias

The last main threat that is being discussed in the DREAM study and is observed in articles within this review comprises causality bias, where a proposed cause–effect relationship does not need to represent the truth.

Another specific type of causality bias that is important to consider is reverse causation, also primarily due to the long latency period of AD. This is a scenario where the association is distorted by a reversal of the perceived exposure and outcome. In such a case, AD might rather increase the risk of developing HZ instead of the other way around. Emphasizing the importance of a time-window to limit such a reverse causality bias, Linard et al. introduced a lag-time of one year from the index date to the first prescription of AD medication, during which no AD medication may be prescribed [19].

The DREAM study framework provides practical methodological solutions to address the biases identified in the reviewed studies. Specifically, DREAM advocates for lag-time analyses to minimize reverse causality, better exposure definitions to reduce misclassification, and advanced statistical methods to adjust for unmeasured confounding. Applying these strategies systematically in future observational studies could substantially improve the reliability of research investigating viral exposures and dementia risk.

## 10. Conclusions

This systematic review suggests that VZV vaccination, especially against RZV, and antiherpetic drugs (AHDs) may help reduce the risk of dementia and AD by preventing viral reactivation and related neuroinflammation. However, the evidence on VZV infection and the risk of AD is mixed, with studies reporting both increased and decreased risk, likely influenced by antiviral treatment or population differences. These inconsistencies underscore the complex link between viral infections and neurodegeneration. To confirm these findings and assess causality, randomized controlled trials are needed to compare cognitive outcomes across vaccinated and unvaccinated individuals, as well as AHD users and non-users, while accounting for confounding factors.

## Figures and Tables

**Table 1:** The included studies in the systematic review.

Sample Size	Study Design	Region	Year	Study
229,594	Retrospective cohort	South Korea	2021	Bae et al. [18]
265,172	Retrospective cohort	Sweden	2021	Lindman et al. [24]
281,102	Retrospective cohort	South Korea	2022	Shim et al. [26]
1,483,195	Retrospective cohort	Denmark	2022	Schmidt et al. [25]
1,125,691	Case–control	South Korea	2021	Choi et al. [23]
143	Retrospective cohort	United States	2022	Lehrer et al. [27]
212,417	Retrospective cohort	United States	2023	Harris et al. [31]
228,223	Case–control	United Kingdom	2021	Lophatananon et al. [28]
136,016 (VHA); 172,790 (MarketScan)	Retrospective cohort	United States	2021	Scherrer et al. [29]
336,341	Retrospective cohort	Wales	2022	Schnier et al. [30]
68,291	Retrospective cohort	France	2022	Linard et al. [19]

**Table 2:** VZV infection and AD risk.

Study	Year	Region	Sample Size	Age (Years)	Adjusted for Confounders?	Main Findings
Bae et al. [18]	2021	South Korea	229,594	≥50	Yes (age, sex, comorbidities)	HZ infection associated with increased AD risk (aHR = 1.12; 95% CI: 1.04–1.19).
Lindman et al. [24]	2021	Sweden	265,172	≥50	Yes (age, sex, comorbidities)	Untreated herpes infections increased AD risk (aHR = 1.50; 95% CI: 1.29–1.74).
Shim et al. [26]	2022	South Korea	281,102	≥50	Yes (age, sex, comorbidities)	HSV infection increased AD risk (aHR = 1.18; 95% CI: 1.16–1.20); VZV infection increased risk (aHR = 1.09; 95% CI: 1.07–1.11).
Schmidt et al. [25]	2022	Denmark	1,483,195	≥40	Yes (age, sex, comorbidities)	HZ infection associated with decreased AD risk (HR = 0.93; 95% CI: 0.90–0.97).
Choi et al. [23]	2021	South Korea	1,125,691	≥60	Yes (age, sex, comorbidities)	No association between HZ infection and AD (OR = 0.90; 95% CI: 0.84–0.97).

The odds ratios and hazard ratios from different study designs are not directly comparable.

**Table 3:** VZV vaccination and AD risk.

Study	Year	Region	Sample Size	Age (Years)	Vaccine Type	Adjusted for Confounders?	Main Findings
Lehrer et al. [27]	2022	United States	143	≥60	Zostavax	Yes (age, sex, comorbidities)	Zostavax associated with 15% reduction in AD risk.
Harris et al. [31]	2023	United States	212,417	≥65	Zostavax, Shingrix	Yes (age, sex, comorbidities)	Zostavax reduced AD risk (RR = 0.92; 95% CI: 0.90–0.94); Shingrix reduced AD risk (RR = 0.27; 95% CI: 0.25–0.29).
Lophatananon et al. [28]	2021	United Kingdom	228,223	≥70	Zostavax	Yes (age, sex, comorbidities)	Vaccination reduced AD risk (OR = 0.91; 95% CI: 0.89–0.92).
Scherrer et al. [29]	2021	United States	136,016 (VHA); 172,790 (MarketScan)	≥65	Zostavax	Yes (age, sex, comorbidities)	Vaccination reduced AD risk (aHR = 0.75, 95% CI: 0.71–0.80 in VHA cohort; HR = 0.70, 95% CI: 0.55–0.88 in MarketScan cohort).
Schnier et al. [30]	2022	Wales	336,341	≥70	Zostavax	Yes (age, sex, comorbidities)	Vaccination reduced AD risk (aHR = 0.81; 95% CI: 0.77–0.86).

The odds ratios and hazard ratios from different study designs are not directly comparable.

**Table 4:** AHD use and AD risk.

Study	Year	Region	Sample Size	Age (Years)	AHD Type	Adjusted for Confounders?	Main Findings
Lindman et al. [24]	2021	Sweden	265,172	≥50	Acyclovir, Valacyclovir	Yes (age, sex, comorbidities)	Antiviral treatment reduced AD risk (aHR = 0.89; 95% CI: 0.86–0.92).
Linard et al. [19]	2022	France	68,291	≥65	Acyclovir, Valacyclovir	Yes (age, sex, comorbidities)	AHD use reduced AD risk (HR = 0.85; 95% CI: 0.75–0.96).

The odds ratios and hazard ratios from different study designs are not directly comparable.

## Data Availability

The original contributions presented in this study are included in the article. Further inquiries can be directed to the corresponding author.

## Abbreviations

AD	Alzheimer’s Disease
Aβ	Amyloid-beta
AHD	Antiherpetic Drug
CI	Confidence Interval
CNS	Central Nervous System
DREAM	Drug Repurposing for Effective Alzheimer’s Medicines
HR	Hazard Ratio
HSV	Herpes Simplex Virus
HZ	Herpes Zoster
MeSH	Medical Subject Headings
OR	Odds Ratio
RCT	Randomized Controlled Trial
RR	Relative Risk
RZV	Recombinant Zoster Vaccine
SAIL	Secure Anonymised Information Linkage
TNF	Tumor Necrosis Factor
VHA	Veterans Health Administration
VZV	Varicella Zoster Virus
